# Epidural Fat and Perineural Adipose Tissue Septic Emboli Mimicking Peripheral Nerve Sheath Tumor in a Dog

**DOI:** 10.1155/2022/9173442

**Published:** 2022-12-06

**Authors:** Yael Merbl, Kelly M. Ramsay, Ashley Hanna, Annie V. Chen, Laura Anne White, Claire R. Burbick

**Affiliations:** ^1^Department of Clinical Sciences, College of Veterinary Medicine, Cornell University, Ithaca, NY 14853-6401, USA; ^2^VDx Veterinary Diagnostics and Preclinical Research Services, Davis, CA 95616, USA; ^3^Department of Veterinary Clinical Sciences, College of Veterinary Medicine, Washington State University, Pullman, WA 99164-7060, USA; ^4^Washington Animal Disease Diagnostic Laboratory, College of Veterinary Medicine, Washington State University, Pullman, WA 99164-7060, USA

## Abstract

*Summary*. A 9-year-old 35.6 kg (90 lb) female neutered German Shepherd dog was admitted due to progression of tetraparesis. The dog presented pyrexia, mild leukocytosis, and nonambulatory tetraparesis with decreased general proprioception and withdrawal in all the limbs, with the front limbs more severely affected. Magnetic resonance imaging revealed T2-weighted image (WI) hyperintense, contrast-enhancing lesion at the level of the C6-C8 spinal nerves, and epidural fat, suspected to be an infiltrative neoplasm. Medical treatments during hospitalization included glucocorticoids, antibiotics, and supportive care. Euthanasia was elected 4 days later due to financial constraints, despite clinical improvement. Postmortem findings revealed septic emboli (SE) in the epidural fat exiting the canal and following the tract of the spinal nerve roots and nerves. *Staphylococcus pseudintermedius* was identified as the causative agent. Although the incidence of SE without severe systemic disease is considered low in dogs, it should be considered in the differential diagnosis of focal intraspinal disease.

## 1. Case History

A 9-year-old female spayed German Shepherd dog was presented for evaluation of an approximately two weeks of history of progressive nonambulatory tetraparesis. The dog was stoic; hence, hyperesthesia region was not recognized. Prednisone (PrednisTab®, PO, 1.1 mg/kg/day) and omeprazole (Med-Vet International, PO, 1 mg/kg/day) were prescribed by the referring veterinarian. The dog showed improvement initially but then deteriorated. The prednisone dose was increased to 1.7 mg/kg/day, yet the dog continued to decline to nonambulatory tetraparesis but maintained eating and drinking. Medical history included prior urinary tract infections (UTIs), right tarsal fracture, and left cranial cruciate ligament tear that were repaired on different occasions by the referring veterinarian several months ago. No further information was described in the medical records with regard to the nature of the UTI.

On admission, the dog was bright, alert, and responsive. Owners reported the dog was eating and drinking normally. She was panting with a temperature of 39.4°C. Her systolic blood pressure was 160 mmHg and her heart rate was 120 bpm. Additional findings included a firm, moveable mass on the left tarsus with significant atrophy of both the hind limbs and a scar tissue on her right tarsus from the previous surgery. Neurological examination revealed nonambulatory tetraparesis, more severe on the right side. General proprioception was absent in both the pelvic limbs and the left thoracic limb and delayed in the right thoracic limb. Spinal reflexes were decreased in all 4 limbs, while the front limbs were more severely affected. No hyperesthesia was noted along the skeletal bones, paravertebral musculature, or spine. The remainder of the exam was unremarkable. Neurolocalization included either a neuromuscular disorder or multifocal lesions involving both intumescences.

Differential diagnoses, based on physical and neurologic examination, included neoplasia (multi focal), infectious or inflammatory disease, or possibly a paraneoplastic syndrome. Degenerative changes such as intervertebral disc diseases or spinal canal stenosis could explain the neurologic deficits noted but would have to include a comorbidity to explain the marked lethargy and pyrexia. Ischemic insults, trauma, and intoxication were also considered less likely.

Complete blood count showed leukocytosis (20.7 × 10^3^ cells/*μ*L; reference range, 4.5 × 10^3^ cells/*μ*L to 16 × 10^3^ cells/*μ*L)consisting of neutrophilia without toxic changes. Other hematologic values were within normal levels (WNLs). Serum biochemical abnormalities included increase alkaline phosphatase (614 U/L; reference range, 0 to 96 U/L) and alanine aminotransferase (242 U/L; reference range, 0 to 100 U/L) activities. All other variables (i.e., electrolytes, hepatic, and kidney functions) were WNL. Urinalysis obtained by cystocentesis, revealed a hazy dark yellow urine with a specific gravity higher than 1.035, 3+ blood, more than 75 RBC/HPF, 6-15 WBC/HPF, with no evidence of casts or crystals, and cocci in aggregates and occasionally chains consistent with an active urinary tract infection (bacterial cystitis).

Cervical radiographs showed a mild narrowing of C4-C5 intervertebral disc space and moderate proliferation of the cervical articular facet joints at C3-C4 and C4-C5. Abdominal and thoracic focused assessment ultrasonography and thoracic radiographs were unremarkable.

The dog was hospitalized with intravenous fluids (PLASMA-LYTE A Injection pH 7.4, Deerfield Illinois, 60015, USA), and due to the fever and bacteriuria, sulfamethoxazole and trimethoprim (Amneal, PO, 15 mg/kg BID) was administered alongside with nursing care. Within the next 2 days, temperature normalized and the dog became ambulatory on three of its limbs, except for the right thoracic limb. MRI was performed at the 3^rd^ day of hospitalization. By that time, marked improvement was noted in all four limbs, the dog had regained ambulatory status, and the spinal reflexes were normal, except for the left front thoracic limb, showing a non-weight-bearing lameness with decreased withdrawal reflexes. An updated neurolocalization of C6-T2 spinal cord, spinal cord segments, or nerves lesion was considered most likely, assuming that the dog either responded to antibiotics or to supportive care.

**Figure 1:**
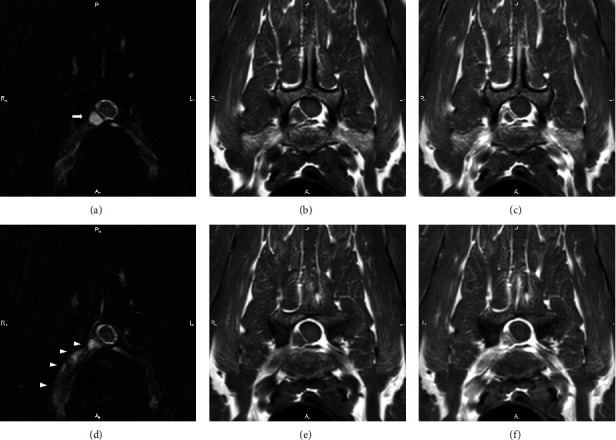
Transverse view of the cervical spine at the level of C8 at the level of nonenhancing material (white arrow), (a) T2 FS Dixon, (b) T1 precontrast, (c) T1 postcontrast, and at the nerve root level (white arrowhead), (d) T2 FS Dixon, (e) T1 precontrast, and (f) T1 postcontrast. There is moderate enlargement, heterogeneity, and contrast enhancement along the right ventral C8 nerve.

MRI (Ingenia 3.0 T MR System, Philips) of the cervical spine revealed heterogeneously contrast-enhancing soft tissue (heterogeneous on T2WI and isointense on T1WI) within the right ventrolateral aspect of the spinal canal from C6 to T1. The tissue was contiguous with the right ventral C8 nerve root (Figure 1) and to a lesser degree with the C7 nerve root, both of which were contrast enhancing and enlarged, expanding to the intervertebral foramina. The tissues surrounding the right C6-7 articular facet were T2 hyperintense and contrast enhancing. Compared to other facets of the caudal cervical spine, this facet was enlarged, resulting in mild spinal cord compression (normal signal of the spinal cord). Additionally, a small focal T2 hyperintense, contrast-enhancing lesion was identified within the left ventral C8 nerve root, without a visualized communication to the right-sided tissue. The findings noted that the cervical spinal canal were possibly compatible with infiltrative neoplasia; however, other differentials including inflammatory or infectious causes. Due to financial constraints emerging from the long supportive care of the dog alongside with the guarded prognosis, euthanasia was elected, at that point, and further diagnostic tests were canceled.

**Figure 2:**
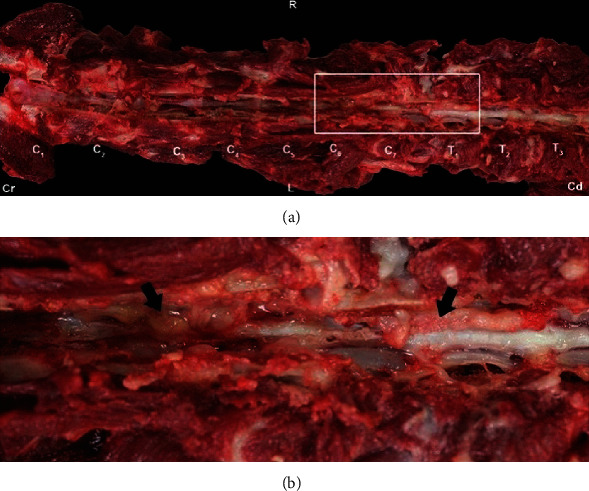
Gross pathology dorsal view of the vertebral column. The dorsal vertebral processes and spinal cord removed, exposing the ventral aspect of the intervertebral canal. (a) Vertebral column from C1 to T3. (b) Vertebral column from C6 to T1, inset of Figure 1(a). Within the right ventrolateral aspect of the intervertebral canal from C6 to T1 are aggregates of pale tan to pink, soft, gelatinous exudate (arrowheads). Exudate extended into the right intervertebral foramina between C5-C6, C6-C7, and C7-T1. Cr: cranial aspect; Cd: caudal aspect; R: right; L: left; C1-T3: approximate locations of each of the cervical and thoracic vertebrae.

On gross postmortem examination (Figure 2), the right ventrolateral aspect of the intervertebral canal from C6 to T11 contained multifocal loosely adhered, plaque-like aggregates of opaque, pale tan to pink, soft, and gelatinous exudate that extended into the right intervertebral foramina between C5-C6, C6-C7, and C7-T1. Both kidneys contained several, dark red foci (5-7 mm), distributed throughout the cortex and the corticomedullary junction, without involvement of the renal pelvis.

**Figure 3:**
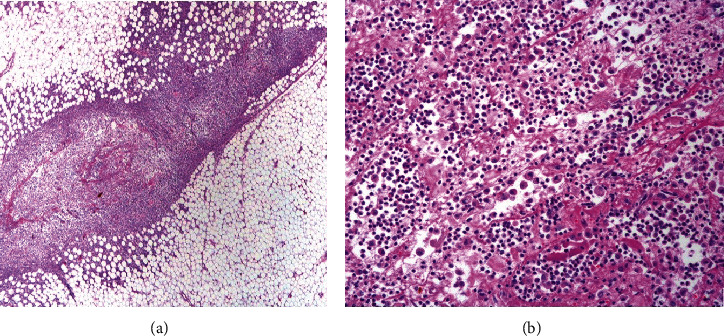
Histopathology micrograph. Intravertebral adipose tissue H&E, region of C6-C8. (a) The intravertebral adipose tissue is variably effaced by inflammation, fibrin, and necrosis. (b) Higher magnification view of the inflammation revealed an infiltrate composed of large numbers of neutrophils and macrophages admixed with abundant fibrin. No pathogens were noted.

Histopathological evaluation revealed suppurative steatitis (Figure 3) with C6-C8 region more severely affected, involving the epidural fat, perineural adipose tissue surrounding the right-sided C7 and C8 nerve roots, and the perimysium of the surrounding muscle. Resident adipose tissue was extensively effaced by inflammation and necrotic debris. Interestingly, the nerve roots themselves were unaffected. No pathogens were identified with special stains including the Brown-Hopps Gram, Fite's acid fast, Grocott's methenamine silver, Giemsa, or periodic acid-Schiff.

**Figure 4:**
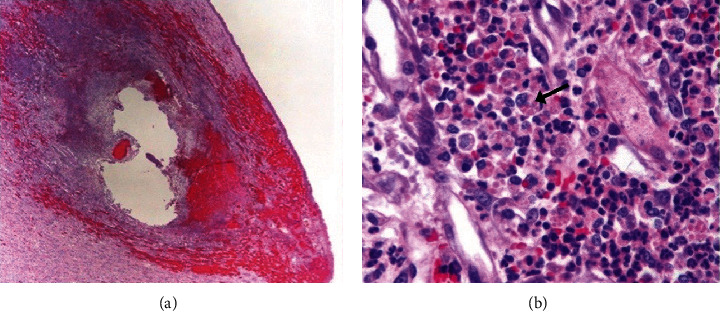
Histopathology micrograph, Kidney H&E. (a) Within the grossly red renal foci, the renal medulla was multifocally effaced by suppurative foci. (b) Higher magnification view of the infiltrating neutrophils and macrophages, the latter of which often contained granular to globular, eosinophilic, intracytoplasmic phagocytic debris (arrow). No pathogen were noted.

The renal parenchyma (Figure 4) was multifocally effaced by inflammation and necrosis, similar to that seen in the epidural adipose tissue. No pathogens were observed with the same set of stains performed on the epidural fat. No overt routes of bacterial entry were identified.

All gross and histologic findings, associated with the right tarsal fracture and left cranial cruciate ligament tear that were repaired, were deemed as within limits for normal scarring responses, without any signs of previous or current infection.

Bacterial identification from pooled liver, kidney, and spleen tissues was performed using a matrix-assisted laser desorption time-of-flight mass spectrometer (MALDI-TOF MS, Bruker Daltonics, Billerica MA, USA) which identified the isolate as *Staphylococcus pseudintermedius*.

## 2. Discussion

The histological findings in this case were compatible with a septic process leading to depositions of SE within the kidneys, vertebral canal, and adipose tissue. Septic embolus is a class of early or late infectious disease complication [1]. SE have been previously reported in veterinary medicine [2], usually as a sequelae of systemic disease such as infective endocarditis or septicemia [3]. The kidneys, spleen, brain, and skeletal muscles are the most common sites of SE in dogs [3]. In our case, the perineural adipose tissue and kidneys were affected. There was no infectious endocarditis, and the origin of the septic emboli has been speculated to arise from the bacteriuria or the kidneys. Unfortunately, since the dog was euthanized, no further diagnostic tests were performed to indicate the origin of bacteriuria.

Bacterial infections within the extradural space of the spinal canal (spinal abscess) as well as subdural empyema (infection causing accumulating purulent material between the dura and the arachnoid meningeal layer) have been reported in small animals (myelopathies secondary to the empyema) [4–10]. A previous report has been published, describing the MRI findings of a spinal empyema, with the epidural lesions appearing as high or mixed signal space occupying lesions in T2WI [3]. Additionally, two possible patterns of enhancement of the extradural material on postcontrast T1WI were described, consisting of either mild to moderate peripheral enhancement or a diffuse pattern of enhancement. Interestingly, all dogs included in that study showed increased signal in gray matter at the site of the lesion on T2W images. In the current case report, the signal and enhancement characteristics of the epidural lesion were similar to those previously described, with regions of diffuse and peripheral enhancement. Based on the histologic findings, the regions of peripheral enhancement may have corresponded to the areas of necrosis seen postmortem. In contrast to the previous report, this case demonstrated no signal changes to the spinal cord parenchyma despite the compressive material in the extradural space. The other relatively unique imaging feature of this case was the lesion extension through the intervertebral foramina which involved the adipose tissue surrounding the nerve roots and spinal nerves. Involvement of the nerve roots has been previously reported in suspected direct inoculation, such as discospondylitis or a bite wound [11, 12]. However, this is the first time that a heterogenous route of infection results in such a long lesion extension through the intervertebral foramina. During necropsy, the nerve roots were unaffected, although the surrounding tissue was inflamed/infected resulting in the imaging appearance. Similarly, multifocal lesions were detected within the kidneys.

*Staphylococcus pseudintermedius* was isolated from the tissue pool. The lesions isolated within the kidneys, and the presumed embolic pathogenesis of the spinal lesion, make *Staphylococcus pseudintermedius* the most likely cause of the septic infection. *Staphylococcus pseudintermedius* is a ubiquitous skin bacterium and was previously implicated in surgery implant infections [13]. Despite a thorough histopathological examination of different body tissues and the area of prior surgeries, the source of our infection was not identified; however, the dog had a UTI. Since urine culture was not performed and if it was speculated about the same bacteria as the causative agent of the infection, it is reasonable to consider the UTI as the source of infection.

Nerve root enlargement is the most common abnormality detected on MRI, in neoplastic processes, such as lymphoma or peripheral nerve sheath tumor. The differentiation of the normal nerves from the surrounding inflamed/infected tissues was not applicable based on MRI. A previous report supported the use of a fine needle aspiration (FNA) to differentiate spinal nerve neoplasm and inflammatory processes [14]. In the current case, FNA of the lesions could have been helpful in supporting an inflammatory/infectious etiology. FNA or full thickness tissue biopsy/excisional biopsy in cases that do not respond to treatment may allow definite diagnosis but carries potential complications.

SE should be included in the differential list for focal nerve enlargements especially in animals presenting with fever and leukocytosis, even without concurrent indication of endocarditis and/or fulminant septic condition. MRI findings that may support diagnosis include contrast enhancement of adjacent tissues and nonuniform enhancement of the tissue within the spinal canal and associated with the nerve roots.
